# Ion Chromatography and Related Techniques in Carbohydrate Analysis: A Review

**DOI:** 10.3390/molecules29143413

**Published:** 2024-07-20

**Authors:** Rajmund Michalski, Joanna Kończyk

**Affiliations:** 1 Institute of Environmental Engineering of Polish Academy of Sciences, 41-819 Zabrze, Poland; 2 Institute of Chemistry, Faculty of Science & Technology, Jan Dlugosz University in Czestochowa, 42-200 Częstochowa, Poland; j.konczyk@ujd.edu.pl

**Keywords:** carbohydrates, ion chromatography, environment, biomass, plant

## Abstract

Ion chromatography and related techniques have been the most popular separation methods used in the determination of organic and inorganic anions and cations, predominantly in water and wastewater samples. Making progress in their development and introducing new stationary phases, methods of detection and preparation of samples for analyses have given rise to the broadening of their analytical range. Nowadays, they are also used for substances that are not ionic by nature but can convert to such forms under certain conditions. These encompass, among others, carbohydrates, whose role and significance in humans’ lives and environment is invaluable. Their presence in the air is mostly due to the industrial burning of biomass for energy production purposes. In addition, the content of sugars in plants, fruits and vegetables, constituting the base of human diets, affects our health condition. Given that, there is not only a need for their determination by means of routine methods but also for searching for novel analytical solutions. Based on literature data from the past decade, this paper presents the possibilities and examples of applications regarding ion chromatography and related techniques for the determination of carbohydrates in environmental samples, biomass and plants constituting food or raw materials for food production. Attention has been paid to the virtues and limitations of the discussed separation methods in this respect. Moreover, perspectives on their development have been defined.

## 1. Introduction

It is estimated that the number of chemical compounds present in the direct vicinity of humans—depending on the concentrations at which these substances start to be considered present—ranges from approximately 100,000 up to 10 million. Among them, only a minute percentage is constituted by the ones that can be considered neutral or harmless to humans’ health. Due to the roles played in the human body and the significance of many representatives, it is necessary to monitor these substances in accordance with the regulations in force. Carbohydrates (saccharides, sugars) belong to this group of compounds. Bearing in mind their chemical structure, they are divided into simple sugars (monosaccharides) and compound sugars (oligo- and polysaccharides). They constitute the basic building and energetic material of plant and animal cells, being absolutely essential for the normal functions of living organisms. These compounds are mostly known as nutritional ingredients, existing naturally and supplying the body with energy. In turn, the energy has a fundamental significance in maintaining optimum functions of the brain, the muscles and the nervous system. Plants, animals, bacteria and algae can be the source of sugars [1]. Using carbon dioxide and water, plants are able to synthesize simple sugars in the process of photosynthesis. Conversely, the remaining organisms take them up, first and foremost, from food. The knowledge of a particular plant’s sugar profile, either as a whole or as its given part (the root, the stem, leaves, fruits, seeds), is pivotal in terms of using it in various technological processes, particularly the ones associated with the manufacture of food or drugs. Due to increasing consumer health problems, resulting from sugar intolerance or abnormal metabolism, knowledge on the content of these compounds in raw materials and foodstuffs has become absolutely required for elaborating proper nutritional schedules.

On the other hand, qualitative and quantitative carbohydrate analysis in diverse environmental matrices enables scientists to define the anthropogenic sources of these compounds. To date, studies have been published discussing carbohydrates present in surface waters [2], snow [3], wastewater [4], soils [5], sediments [6] and tree and herbaceous biomass [7]. However, the greatest attention has been paid to the analysis of these compounds in the samples of atmospheric aerosols. An interest in the air-contained sugars stems from the global politics encouraging the burning of a good deal of biomass as “renewable energy”. This kind of approach is the subject of serious controversies [8], as it increases the air pollution not only with carbon dioxide but also with other dangerous substances that may exert an adverse effect on humans’ health. Primary and secondary organic aerosols are ubiquitous. Their share in the air pollution depends on the sources of emission, including the type of biomass and conditions of its burning, and on atmospheric conditions. Dusts present in them, with particle diameters equal to or less than 10 µm, may penetrate the human respiratory and circulatory systems, and, as a result, lead to pulmonary and cardiac damage. Due to the burning of plants rich in cellulose and hemicellulose, many low-molecular-weight sugar compounds are formed, e.g., sugar anhydrides, simple sugars and sugar alcohols [7]. Some sugar anhydrides are biomass burning markers enabling scientists to determine the anthropogenic sources of aerosols in the atmosphere. Their representatives are as follows: levoglucosan (1,6-anhydro-β-d-glucopyranose), mannosan (1,6-anhydro-β-d-mannopyranose) and galactosan (1,6-anhydro-β-d-galactopyranose). The first is generated during a thermal breakdown of cellulose; the other two are formed due to pyrolytic degradation of hemicellulose [9,10]. In turn, the presence of sugar alcohols, e.g., arabitol and mannitol, and trehalose in aerosols is connected with the presence of fungal spores. On the other hand, the content of glucose, fructose and/or sucrose in aerosols is most probably derived from the presence of microorganisms, algae or fragmented pollens and flower buds [11,12].

The analysis of sugar content in environmental samples and in food is based on a variety of techniques and analytical methods, including ion chromatography (IC) and related methods. This paper is aimed at reviewing literature data in the scope of this sort of analyses, with a focus on detailed separation conditions.

To date, no detailed summary of experimental data in this area has been published. The review papers available in the literature usually concern the comparison of different methods (colorimetric, spectroscopic, chromatographic, electrophoretic) or specific chromatographic methods for the analysis of sugars, mainly in food samples [13,14,15,16,17]. A small number of review papers have compiled data on sugar analytics in environmental samples. In paper [18], the authors gave examples of analyses that allow the determination of concentrations of sugar anhydrides (levoglucosan, glucosan and mannosan) in environmental samples by gas and liquid chromatography, and capillary electrophoresis. Boshagh [19] reviewed the methods of carbohydrate determination in the dark fermentative hydrogen production process, and Faixo et al. [20] included the analysis of sugars when reviewing methods for the analysis of biologically resistant organic components of sewage sludge and wastewater.

## 2. Methods of Carbohydrate Determination

A number of physical and chemical methods, both classic (manual) and instrumental, are used for the determination of carbohydrates [21]. Physical methods include densitometric, polarimetric and refractometric approaches. They are approximate, semi-quantitative methods used mostly for determining water-soluble sugars [22]. Chemical methods of sugar analysis cover titrimetric and colorimetric solutions. The former are non-specific and exploit the reducing properties of carbohydrates. The latter are based on the measurement of absorbance for colorful compounds synthesized by chemical reactions between sugars and multiple chemical reagents. The most common instrumental methods used in the analysis of sugars are electrophoretic [23] and chromatographic [24] methods, with a predominance of high-pressure anion exchange chromatography (HPAEC) [25] and ion chromatography (IC) [26,27]. Also, but to a smaller degree, other chromatographic techniques are used for the analysis of carbohydrates, i.e., gas chromatography (GC) [28,29], thin-layer chromatography (TLC) [30] and hydrophilic interaction liquid chromatography (HILIC) [31]. However, they are labor-intensive due to the necessity of proper preparation of a sample, e.g., by converting an analyte into its volatile forms in the course of the derivatization reaction [32]. In turn, the lack of chromophore or fluorophore groups in the molecules of sugars makes their direct detection impossible by means of spectrophotometric or fluorescence detectors commonly used in liquid chromatography [33]. IC originates from high-pressure liquid chromatography (HPLC). Both of them belong to the group of liquid chromatography (LC) methods. On that basis, they are sometimes treated as synonyms. Apart from nomenclature-related differences between HPLC and IC, there are also others, connected to the construction of the chromatographic system and mechanisms underlying the separation of sample constituents. IC, as a variant of liquid chromatography, requires the use of the same or similar parts of the chromatographic system (pumps, injectors, detectors) as the ones used in HPLC. However, if using IC and HPLC (particularly HPAEC) notions interchangeably, it should be remembered that there exists a diversity of implemented eluents (mobile phases), analytical columns and detection methods. Therefore, the modes of separation are based on distinct phenomena. From a formal point of view, a typical HPLC chromatograph is not an ion chromatograph, due to the fact that, among other things, a metal pump head used with it should not be used with IC due to the risk of washing metals off, for example when analyzing metal or metalloid ions. At present, all the ion chromatograph components, e.g., a pump head, that eluent and analyzed samples come to a direct contact with, are fully made of inert fabrics such as polyether-ether ketone (PEEK). In this paper, the HPAEC and IC terms are used interchangeably, where justified.

### 2.1. High-Pressure Anion Exchange Chromatography (HPAEC)

Początek formularzaHPAEC allows for the qualitative and quantitative analysis of sugars containing up to 70 carbon atoms, due to the presence of electrochemically active hydroxyl groups in their molecules. In the basic solutions, neutral and acidic mono- and oligosaccharides are subject to the reaction of electrolytic dissociation with the formation of anionic forms, which are separable in anion-exchange columns. Aqueous solutions of sodium and potassium hydroxides in a broad range of concentrations, i.e., from several to hundreds of mmol/L, are the most commonly used eluents. Hydroxide ions (OH^−^) play the role of eluting ions and define the mobile phase pH value. A change in their concentration in the mobile phase considerably affects their retention times. While the dissociation of carbohydrates, and, consequently, retention times, increase along with an increase in pH values, a resulting higher concentration of eluent ions causes the shortening of retention times. These two effects compensate each other unless carbohydrates are fully dissociated. In the case of complete dissociation, a further increase in OH^−^ levels only leads to the shortening of retention times. Carbohydrates with a high affinity to the stationary phase may be separated by the addition of sodium acetate (NaOAc) to the eluent. This salt accelerates the elution of analytes, strongly bound to the stationary phase, and provides a better control of selectivity. Since the optimum pH for such a system is 13, the concentration of hydroxide solution is usually maintained at a constant 0.1 mol/L. Meanwhile, during the separation process, increasing amounts of sodium acetate are added to the hydroxide solution. When it comes to preparing hydroxide-based eluents, special attention should be paid to ensuring that the eluents are free from carbonates, as the presence of even small amounts of them worsens separability and changes retention times. Eluents should be prepared from a concentrate. Deionized water used for the preparation of the mobile phase must be precisely degassed, e.g., with helium.

An analytical column, specifically a stationary phase used within it, is the “heart” of every chromatographic system. A list of the most popular marketed ion-exchange columns used for the separation and determination of different types of sugars and their derivatives is presented in Table 1. As can be seen in Table 1, the offerings of companies selling columns for the analysis of carbohydrates are not plenteous. Nevertheless, the stationary phases offered are differentiated enough to allow chemists a proper choice of a column for a given type of carbohydrate and sample matrix. The Thermo Scientific™ (Waltham, MA, USA) (former Dionex™, Sunnyvale, CA, USA) CarboPac™ PA1 column, introduced into the market in 1983, is the oldest analytical column designed for carbohydrate determination. Since its stationary phase is not mechanically stable, the column has to work at a relatively low flow rate of an eluent. Dionex CarboPac PA10 and PA20 columns are usually used for the separation of diverse neutral and acidic mono- and disaccharides. The Dionex CarboPac SA10 column, designed for a quick mono- and disaccharide analysis in biofuels and food, is the newest addition to the family of Dionex CarboPac columns.

Apart from commercially available columns, specially prepared stationary phases can be optionally used for carbohydrate determination. Guo et al. [34] synthesized a stationary phase based on polystyrene-divinylbenzene-glycidyl methacrylate grafted with poly (amidoamine) dendrimers (PAMAM). They implemented it as stationary phase for a column used for the separation of four sugars (trehalose, glucose, maltotriose and galacturonic acid) in a variety of sample types. In that particular column, polysaccharides of long retention times are successfully separated in the presence of monosaccharides. In turn, Liu et al. [35] offered stationary phases based on poly (glycidylmethacrylate (GMA)-divinylbenzene (DVB) microspheres) layers as beds of columns dedicated for a simultaneous separation of inorganic ions and carbohydrates. Such a column of high capacity (0.366 mM/column) allowed for the separation of five carbohydrates (arabinose, fucose, glucose, maltose and maltotriose) from the mixture of their standards. Stationary phases obtained by Zhao et al. [36], based on sucrose with quaternary ammonium groups as functional groups, turned out to be effective in a concomitant separation of four carbohydrates (fucose, glucose, trehalose and raffinose) as well as numerous organic and inorganic anions.

### 2.2. Ion Chromatography

At present, IC is the most popular instrumental technique for the analysis of a broad range of anions and cations, both organic and inorganic. Furthermore, it finds applications for determining not only ion compounds but also compounds convertible to these forms. The paramount advantages of IC are reflected in the following aspects: the possibility of simultaneous determination of several ions within a short time; a small amount of sample required for an analysis; the possibility of using different detectors; simple methods of sample preparation, a high selectivity of analyte separation; and, last but not least, safety and low costs of daily use. Given that, as early as the 1980s, IC was acknowledged as a reference method for determination of common inorganic ions in waters and wastewater [37]. The most important varieties of IC are ion exclusion chromatography (IEC) [38] and ion pair chromatography (IPC) [39]. IEC finds its applications mostly in the separation of a broad range of small, neutral or partly ionized molecules, such as carboxylic acids, among others [40]. In turn, IPC utilizes the same types of stationary and mobile phases as in reversed-phase liquid chromatography (RP-LC). The greatest benefits can be taken from hyphenated techniques, which offer a combination of advantages from separation and spectroscopic methods, e.g., IC/ICP-MS. They are useful particularly in the speciation analysis of metals and metalloids [41].

The most important applications of IC regard the determination of the main inorganic anions (e.g., F^−^, Cl^−^, NO_2_^−^, NO_3_^−^, Br^−^, PO_4_^3−^, SO_4_^2−^) and cations (e.g., Na^+^, K^+^, NH_4_^+^, Ca^2+^, Mg^2+^) in all types of waters, wastewater and food [42,43,44]. New kinds of stationary phases of various separation mechanisms, as well as new methods of sample preparation and detection, enable scientists to broaden the range of the aforementioned applications to essentially all ionic and ionogenic substances, including carbohydrates. The paramount parameters deciding the efficiency of the separation and determination process in IC are as follows: variant of packing in an analytical column; eluent type, concentration, pH and flow rate; detector type and its operation parameters; injection volume and sample matrix; and a method of sample preparation for the analysis.

IC utilizes various detectors, depending on a type of sample matrix or a type of analyte [45,46]. Conductometric and spectrophotometric detectors are the most common in IC. However, they are useless at determining carbohydrates. From among detection methods used in LC-aided determination of sugars, pulsed amperometric detection (PAD) predominates. It is based on the measurement of electric current appearing in a redox process of the analyte molecules on the surface of working electrode at a defined potential. An amperometric detector has a series of advantages, i.e., high detectability (from 10^−7^ to 10^−15^ g depending on the nature of a given compound); a small chamber volume (0.01–5 μL), making it possible to work with columns of facultative size; a simple construction; and a relatively low cost of operation. In 1983, Rocklin and coauthors were among the first to release works on the use of the IC-PAD system for the analysis of carbohydrates [47]. Following that, the potential of its applications was discussed in detail by Johnson in 1986 [48]. This particular publication is considered a milestone in the development of the HPAEC-PAD technique. Other detectors used in IC for the determination of carbohydrates are the mass spectrometer detector (in a series of configurations) [49] and evaporative light scattering detector (ELSD) [50].

The separation of sugars with the IC technique can be based on one of four mechanisms, i.e., gel filtration, ion exchange, distribution and adsorption. Historically, gel filtration was the first mechanism used for the separation of carbohydrates. In spite of long retention times of analytes and poor resolving power, this method allowed for the separation of carbohydrates in accordance with their molecular masses. At present, it is mainly used for the analysis of polysaccharides [51]. Even though analytes are characterized by relatively long retention times, a typical ion exchange is more and more often used in the analysis of carbohydrates. This is mainly because of low prices of mobile phases. In turn, in order to separate mono- and oligosaccharides, the separation mechanism uses stationary phases with bonds, e.g., amine, in combination with eluents, e.g., based on water–acetonitrile, where the concentration of acetonitrile allows for the control of carbohydrate retention. On the other hand, adsorption chromatography is rarely used for the separation of carbohydrates, as it requires a previous sample derivatization.

An analyzed sample standard is a key causative factor responsible for the separation efficacy of chromatographic peaks corresponding to respective constituents. Analytical systems and parameters suggested in the literature for standard solutions, i.e., carbohydrate mixtures of simple matrices, are most often HPAEC systems with PAD detection and isocratic elution of a NaOH solution [35,52,53]. Slightly different conditions of carbohydrate analysis were offered by Zhao et al. [36]. They carried out a quantitative determination of fucose, glucose, trehalose and raffinose in a standard mixture, utilizing IC with a damped conductometric detection system and non-commercial column dimensions of 4.6 × 150 mm. The column’s packing contained hydrothermal carbonaceous spheres functionalized with quaternary ammonium groups. Moreover, 5 mM NaOH was used as an eluent. In case samples of more complex matrices, such as environmental, foodstuff, pharmaceutical or biological samples, are taken under analysis, the use of chromatographic conditions offered for the analysis of carbohydrates in standard solutions usually does not bring about satisfactory separation effects of carbohydrate components.

## 3. Carbohydrate Analysis in Different Matrices

### 3.1. Environmental Samples

Environmental samples constitute the second most popular scope of IC use in carbohydrate studies [54], just after food analysis [44,55,56,57,58]. This is particularly the case for atmospheric samples, in that levoglucosan, mannosan and galactosan, among others, are applied to be tested [10]. These are sugar markers of biomass burning. Between 2013 and 2023, as many as 663 papers on levoglucosan determination in atmospheric aerosols were published in the Scopus base (searched terms: “levoglucosan and aerosols”). That represents 2.6 times greater interest in this subject matter compared to the preceding period of 2002–2012 (264 papers). Table 2 presents the last decade’s examples from the literature for the determination of selected sugars in air aerosol samples.

Apart from HPLC and GC, sugars in the air samples were analyzed by using HPAEC equipped with amperometric or mass detection. HPAEC-PAD studies were most frequently conducted in single-column systems with a CarboPac MA1 column [59,60,61,72,73,74,75,76]; however, other systems with different stationary phases also occurred. Atzei et al. [62] used a CarboPac PA20 column, while Makkonen et al. [63]; Clemente et al. [64] and Saarnio et al. [68] used a CarboPac PA10 column. Noura et al. [65] and Samake et al. [66] for the determination of sugars in atmospheric dusts suggested bicolumn (CarboPac MA1 + CarboPac PA1) or tricolumn (Metrosep A Supp 15 + Metrosep Carb 2 + Dionex CarboPac MA1) systems, respectively. In turn, in the system with a mass detector, the following columns were used: CarboPac PA1 [67], CarboPac RPA1 [77] and CarboPac PA10 [63,71]. For the analysis of sugars in air aerosols, Barbaro et al. [69,70] used a bicolumn system composed of CarboPac PA10 and CarboPac MA1 subunits. Most often, the tests were performed in a gradient elution mode, using eluent in the form of NaOH solution or a mixture of sodium hydroxide and sodium acetate. Isocratic elution, with the use of 250 mM NaOH as an eluent, was an adequate method for the quantitative analysis of levoglucosan and its separation from other sugar components of the PM2.5 [73,74] and also PM1 and PM10 dusts [64]. San Rodriguez et al. [77] determined levoglucosan, mannosan and galactosan in air samples, using an IC system with electrospray lithium cationisation and a triple quadrupole tandem mass spectrometer as a detector, a CarboPac RPA1 column and gradient flow of KOH solution. A high-performance anion-exchange chromatograph with a particle-into-liquid sampler and a mass spectrometer (PILS–HPAEC–MS) was also a good instrument for atmospheric sugar markers [68].

A strict relationship between the presence (and quantity) of levoglucosan and other biomarkers in air aerosols and their source (the type of burnt biomass) generates the necessity of carbohydrate determination in the biomass itself, as well. As a rule, sugar profiles of wood derived from various tree species are determined. However, the carbohydrate source may be reflected in plant debris such as pomace, seeds, pips or straw (Table 3).

HPAEC with amperometric detection, a CarboPac PA20 column and gradient elution of sodium hydroxide and acetate mixture is the most common system used for the analysis of carbohydrates obtained from the wood of diverse tree species. This particular method was used to test carbohydrates present in beechwood [78,79,80], hardwood [81,82] and aspen wood [83,84]. Isocratic elution with 10 mM NaOH in the role of an eluent was sufficient for the separation and quantitative determination of levoglucosan, arabinose, galactose, glucose and xylose in the samples of pyrolytic oils from pine tree biomass [85]. In turn, Diaz-Arenas et al. [79] and San Rodriquez et al. [78] proposed an HPAEC-QqQ-MS system with Dionex AminoTrap and CarboPac PA200 columns for the analysis of glucose, arabinose, xylose and glucuronic acid, and xylooligosaccharides originated from enzymatically cleft beech wood, respectively. UV spectrophotometric detection (wavelength 328 nm) used by Lorenz et al. [86] in the HPAEC system with a CarboPac PA200 column enabled the researchers to define sugar profiles in the following materials: pine and beech pulps, the wood of six tree species, wheat straw and sugar cane pomace.

Notably, a proper sample preparation utilizing reductive amination after two-stage acid hydrolysis was required. A classic HPAEC-PAD system with a CarboPac PA1 column and isocratic elution of 10 mM NaOH and 1 mM BaCO_3_ was effective in determining arabinose, galactose, glucose, mannose and xylose in rice husks [87]. A gradient elution mode in an HPAEC system with a CarboPac PA1 column, a suppressor (as an in-line desalter to convert the eluate into an MS-compatible solution) and a mass detector with electrospray ionization in the positive ion mode helped to determine mono- and oligosaccharides in hydrolysates of lignocellulose biomass obtained from sugar cane pomace, wheat and barley straws, and willow wood [88].

Familiarity with sugar profiles of food industry postproduction waste materials enables manufacturers to re-use them in other production processes, e.g., in the manufacturing of food for humans and animals. Remains from the wine industry in the form of grape pomace were investigated using an IC-PAD system merged with a tandem mass spectrometer (MS/MS) [89]. IC made it possible to separate isomers, maintaining a concomitant ability to separate low-molecular-mass neutral oligosaccharides. Oligosaccharides were separated and detected using both an ED- and an MS detector and a CarboPac PA300-4 μm column. Gradient elution was initiated from the mixture of sodium hydroxide/acetate of low concentration, which facilitated the separation of neutral oligosaccharides. Eluent containing higher concentrations of sodium acetate allowed for washing bigger and charged oligosaccharides off the column. Also, the MS/MS tandem made it easier to identify oligosaccharides of a structural nature. Such a system enabled the obtainment of a sugar profile containing 32 oligosaccharides with unique compositions of monosaccharides and 61 oligosaccharide structures, including 30 neutral and 31 acidic structures. In turn, Cardoso de Sa et al. [90], Han et al. [91] and Xie et al. [92] analyzed sugars in bagasse as a potential raw material for the production of ethanol. For that reason, Cardoso de Sa et al. [90] used an HPAEC system containing a reverse pulsed amperometric detector with a glass electrode modified with multi-walled carbon nanotubes containing nickel oxyhydroxide nanoparticles (GCE/MWCNT/NiOOH) as an operating electrode, Pt as an auxiliary electrode and a Ag/AgCl electrode as a reference electrode. Under the conditions of isocratic elution (99% water + 1% 150 mM NaOH at a rate of 1 mL/min), arabinose, galactose, glucose and xylose were separated in 30 min. In order to maintain constant ion strength, a continuous flow of 600 mM NaOH was used in an extender module at a rate of 0.20 mL/min. For the determination of carbohydrates in sugarcane bagasse, Han et al. [91] and Xie et al. [92] used a typical HPAEC-PAD system with isocratic elution of 200 mM NaOH and 244 mM NaOAc, but different columns (CarboPac PA1 and PA20, respectively).

### 3.2. Plants

The presence, amount and type of carbohydrates in plants depends primarily on their species, but also on the growing environment. This knowledge plays an essential role in the use of plants as raw materials in the manufacture of food. Samples of sugar determination in plant materials using IC methods are listed in Table 4.

In order to investigate and compare sugar profiles of various plant species, the following were subjected to studies: whole plants, e.g., *Mesona chinesis* [95,96,97], red deadnettle [101], brown algae [105], Jerusalem artichoke [117], alfalfa [118], and their respective parts, e.g., stems [110], leaves [94,98,108,110,112,113], sprouts [114], roots [94,111], tubers, rhizomes and onions [100], seeds/kernels [99,107,115,116,119], flowers [104], and mucus [102,103]. Data presented indicate that HPAEC-PAD is a method also successfully used for the determination of sugars in plant samples. The use of all types of CarboPac columns and a gradient mode of elution, most commonly using eluent in the form of sodium hydroxide/acetate mixture, allows for the separation of a broad range of sugar components contained in the plant material. However, in some cases, it is sufficient to utilize isocratic elution with eluent in the form of NaOH at diverse concentrations and flow rates [95,96,97,98,105,106,114,115,119]. Apart from columns from the CarboPac series, other columns have also been used to analyze sugars in plants. In order to separate glucose, fructose, xylose, galactose and arabinose from bamboo sprouts, Sun et al. [114] used a Hamilton RCX 30 column in an HPAEC-PAD system with an isocratic flow of 2 mM NaOH and 0.5 mM NaOAc mixture and sample injection volume of 2 mL. Juhari et al. [104] applied IC with amperometric detection, a MetroSep CARB 1 column and isocratic elution (100 mM NaOH) for the analysis of glucose, fructose and sucrose in *Roselle calyx* originated from different geographical regions.

Besides the use of the most popular amperometric detector, plant sugars were detected by means of a mass spectrometer (MS) [93,117], a diode-array detector (DAD) [118] and a UV spectrometer [86,107]. In paper [93], a simple, accurate and sensitive method for a simultaneous determination of 13 carbohydrates in a polysaccharide obtained from *Spirulina platensis* algae is presented. Once properly prepared by ultrasound-enhanced extraction with deionized water accompanied by hydrolysis with 1 M trifluoroacetic acid, samples were subjected to HPAEC analysis in optimized conditions of gradient elution using PAD and MS detectors and a CarboPac PA20 column. In turn, Manns et al. [107] proposed two chromatographic systems for the analysis of carbohydrates in brown seaweeds, i.e., HPAEC-PAD with a CarboPac PA20 column versus borate-HPAEC-UV/VIS (with post-column derivatization by Cu-bicinchoninate at 105 °C and detection at 560 nm) with an Omnifit bore column filled with the strong anion-exchange resin MCI Gel CA08F. They found that the suggested procedure of analysis in the borate-HPAEC-UV/VIS system was more accurate and more repeatable than HPAEC-PAD; however, it could provide detection of merely glucose, xylose and mannose monomers. Meanwhile, the detection of sugar alcohols and uronic acids was possible only in the case of HPAEC-PAD use. Xu et al. [117] also suggested the analysis of sugars in pectins of Jerusalem artichoke in two variants: HPAEC-PAD and HPAEC-ESI-MS, with a sheath liquid interface, a CarboPac PA1 column and gradient elution with NaOH. Their novel interface, enabling the connection of high-pressure anion exchange chromatography (HPAEC) with mass spectrometry equipped with electrospray ionization (ESI), turned out to be a useful tool for the separation, identification and characterization of co-existing sugars in pectin samples, even in trace amounts. The implemented technical solution, such as an addition of protective fluid (50 mM NaOAc in isopropanol with 0.05% acetic acid) into wastewater from an HPAEC column, improves the ESI-MS detector’s sensitivity towards sugars. In this case, it also causes an effective ionization of mono- and disaccharides dependent on the buffer’s concentration and the type of organic solvent. Six sugars and two sugar acids were quantitatively analyzed and effectively separated using the IC-DAD method in the study [118].

### 3.3. Fruits, Vegetables and Fungi

HPAEC with an amperometric detector has also been successfully used for the analysis of carbohydrates in fruits, vegetables and fungi. Examples of such analyses described in the literature are presented in Table 5.

The majority of carbohydrate determinations in the matrices of fruits, vegetables and fungi have been undertaken in the gradient elution mode of various profiles, with the use of most of the columns listed in Table 1. The CarboPac PA100 column was the most commonly used. It enabled scientists to separate sugars occurring in berries [121,122], strawberries [122,135], chickpeas [134] and bananas [137]. Only Pico et al. [132] separated carbohydrates from bean samples in a CarboPac PA100 column using an isocratic elution mode. In the case of fruit sample analyses, mixtures of sodium hydroxide and sodium acetate solutions were applied as eluents. On the other hand, when it comes to vegetables, chickpea sample analysis was carried out in a gradient mode with different NaOH concentrations, and the analysis of bean samples was made by means of an isocratic elution with 10 mM NaOH as an eluent. In order to test chickpeas, besides a CarboPac PA100 column, a CarboPac PA200 column was also applied [134]. The results acquired were compared with the ones measured with the HPLC-RI method. An HPAEC-PAD system with a CarboPac PA100 column allowed for a good separation of glucose, fructose, sucrose, raffinose, stachyose and verbascose within less than 20 min. Moreover, myo-inositol and galactinol were determined both in synthetic standard solutions and real chickpea samples. In the case of the CarboPac PA200 column, separation and quantitative determination of the studied sugars within merely 6 min were possible, with a total working time of 25 min. However, a shift in maximum retention time was observed. It is indicative of a poor repeatability of this method, accounting for an inaccuracy of the quantitative analysis. In turn, the HPLC (SEC)-RI method did not yield satisfactory results, as the peaks were not fully separated and the analysis time period was very long (160 min run time + 30 min washing time).

An HPEAC-PAD system with a CarboPac PA200 column, implemented for the analysis of onion, turned out to be more advantageous than an ultra-high performance liquid chromatographic method using evaporative light scattering detection (UHPLC-ELSD) due to a wider range of quantitative analysis for type S fructooligosaccharides up to the polymerisation level of 18 [130]. Very good degrees of separation and lower detection thresholds and quantification limits were achieved, specifically for higher-molecular-mass saccharides.

Hydro carbonates contained in mulberry fruits [124], noni [125], mirtu [126] and Shiitake fungi [138] were separated in a CarboPac PA1 column with a gradient flow of an eluent. The eluent was either the mixture of NaOH and NaOAc or NaOH alone for fruits and fungi, respectively. A gradient flow of the eluent in the form of NaOH and NaOAc mixture was also used in a CarboPac PA10 column for the determination of sugars and sugar alcohols contained in cherries [129] and red pepper [131]. Under isocratic elution, sugar profiles of custard apple fruits (*Annona squamosa*) [127,128] and apple fruits [136] were determined in different columns, i.e., PA10 and PA20, respectively. The CarboPac PA20 column within the HPAEC-PAD system was also used for the analyses of blackberry [120], bilberry [123] and *Auricularia polytricha* fungi [139]. In turn, John and Luthria [133] utilized the same column in an IC-ESI-MS system with an isocratic elution of water, carrying out an analysis of six sugars, two sugar alcohols and galacturonic acid. Guo et al. [34] suggested a non-commercial PEEK column filled with PAMAM in order to test glucose, trehalose and maltotriose in *Poria cocos* fungi and *Actractylodes macrocephala* herbs. The analytes studied were successfully separated within 5 min in the mode of isocratic elution by means of 10 mM NaOH flowing at a rate of 1 mL/min.

## 4. Limits of Carbohydrate Detection and Quantification

The limit of detection (LOD) and limit of quantification (LOQ) are among the parameters proving that the methodology used is adequate. They must be proper in relation to the norms or requirements defined by legal regulations for a given group of samples, i.e., environmental, pharmaceutical or medical samples, food or raw materials for its production, etc.

LOD and LOQ values, indicated in the literature for a particular carbohydrate determined in various matrices, differ to a quite considerable degree. These differences may result from both the type of analytical method used, the methodology of sample preparation and analysis, and the method of estimating these validation parameters. A number of different possibilities for estimating the LOD and LOQ have been described in the literature, e.g., as three times baseline noise [52], baseline fluctuation and calibration curve slope [99] or based on a regression line [140]. Unfortunately, not all authors provide the values of the LOD and LOQ for the analytical method used, or they do not provide the method of estimating these values, which makes a full comparison of these values impossible.

**Figure 1:**
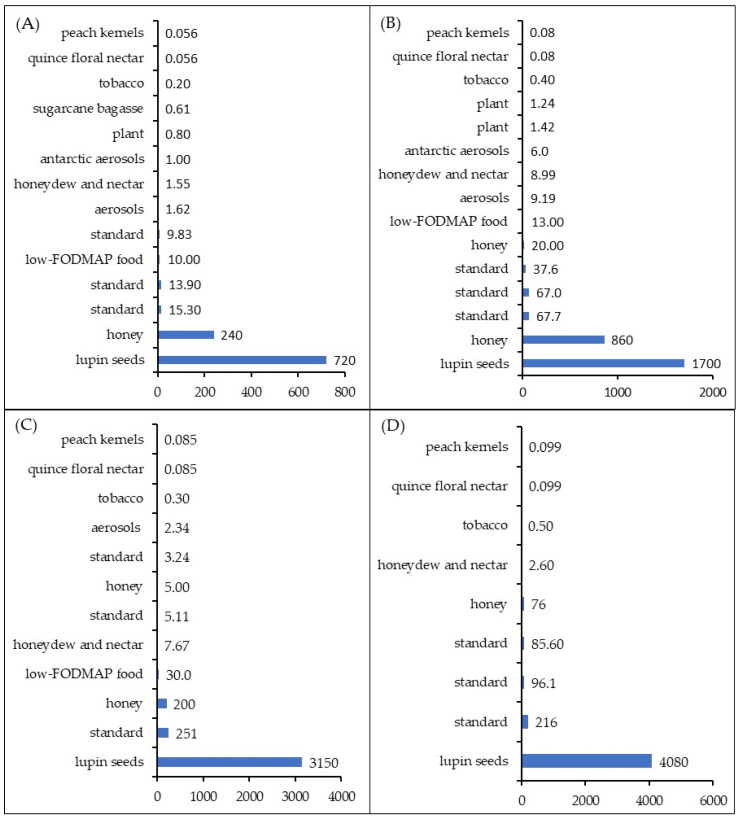
LOD values obtained for glucose (**A**), fructose (**B**), sucrose (**C**) and maltose (**D**) in different matrices [25,52,56,57,65,69,90,98,99,116,140,141,142].

Figure 1 illustrates the LOD values obtained for selected carbohydrates in different matrices. For the most popular and most often analyzed monosaccharides, namely glucose and fructose, the LOD values oscillate from 0.056 µg/L in peach kernels [116] and quince floral nectar samples [140] to 720 mg/L µg/L in lupin seeds [99], and from 0.078 µg/L [140] to 61,700 µg/L in the same samples, respectively, for glucose and fructose (Figure 1A,B). The lowest LOD values of sucrose (0.085 µg/L) (Figure 1C) and maltose (0.099 µg/L) (Figure 1D) were also noted when quince floral nectar and peach kernels samples were subjected to an analysis. In the case of galactose, the lowest LOD (0.0612 µg/L) was found when sugarcane bagasse was studied. The LOD values for sucrose, maltose and galactose in lupin seed samples [99] were considerably higher compared to other matrices, i.e., 3150, 4080 and 740 µg/L, respectively. The literature data clearly indicate that the obtained LOD values are remarkably influenced not only by the analyzed sample matrix itself but also by the types of analyte and detector used.

Barragan and Kubota [52] conducted an HPAEC-PAD analysis of glucose and fructose in the mixture of standards made up of four sugars (glucose, fructose, sucrose and levoglucosan) using the same chromatographing parameters, yet different amperometric detectors, i.e., built of different electrodes. Their findings indicate that lower LOD values are achieved in the case of a Cu|CuO electrode (9.83 µg/L for glucose and 37.6 µg/L for fructose) in comparison with a Au electrode (13.9 µg/L for glucose and 67 µg/L for fructose) and a Cu electrode (15.3 µg/L and 67.7 µg/L for glucose and fructose, respectively). A similar trend was observed when sucrose and maltose were taken under analysis. The obtained LOD values for sucrose (3.24; 5.11 and 251 µg/L for Cu|CuO; Au and Cu electrodes, respectively) were higher than the ones obtained by Zhao et al. [141] for the analysis of sucrose in tobacco (0.4 µg/L). The corresponding LOD values for maltose (85.6; 96.1 and 216 µg/L for Cu|CuO; Au and Cu electrodes, respectively) were higher than the ones acquired in the course of actual sample analyses (Figure 1), excluding the analysis of oligosaccharides separated from lupin seeds [99].

In turn, in their study of sugarcane bagasse biomass samples, Cardoso de Sa et al. [90] used a detector made of a glassy carbon electrode modified with multi-walled carbon nanotubes containing nickel oxyhydroxide nanoparticles (GCE/MWCNT/NiOOH), characterized by LODs for glucose, arabinose, galactose and xylose at the level of several hundred µg/L (198, 375, 270 and 315 µg/L, respectively). Lorenz et al. [143] analyzed carbohydrates in hydrolyzed xylanes using two chromatographic systems, i.e., borate-HPAEC and HPAEC-UV-VIS. These validated methods indicate that it is possible to detect glucose, arabinose, galactose and xylose at considerably lower concentrations based on the method using spectrophotometric detection (0.11, 0.05, 0.06 and 0.05 mg/L, respectively) than with the borane technique (0.42, 1.15, 0.58 and 0.22 mg/L, respectively). Apart from amperometric detectors, mass spectrometry detectors are used for carbohydrate analysis [67,77,142]. MS detectors enable scientists to obtain even lower limits of detection. Levoglucosan analysis in aerosol samples in HPAEC-positive ESI-MS [67] and PILS-HPAEC-MS [68] systems allowed for the detection of this sugar at the levels of 0.4 µg/L and 5–10 µg/L, respectively. Meanwhile, for the same sugar analyzed for atmospheric samples using the IC-TSQ-MS system, LOD values at the level of 0.1 µg/L and lower were obtained [77]. These results favour the MS detector over the standard amperometric detector due to the considerably higher potential for sugar detection presented by the first. For instance, the LOD for levoglucosan determined in PM2.5 dusts with the use of HPEAC-PAD was 30.7 µg/L [74], while MDL (Method Detection Limit) values assessed by Thepnuan et al. [73] and Stracquadanio et al. [76] for the analysis of this sugar in PM2.5 and PM10 dusts amounted to 2.32 and 9.2 ng/m^3^, respectively. Validation of a method conducted in [77] for two variants of calibration, i.e., with and without an internal standard (IS), indicates the possibility of levoglucosan, mannosan and galactosan detection at even lower levels (0.07 (without IS)/0.10 (with IS); 0.12 and 0.5 µg/L, respectively). In turn, the HPAEC-MS method, used by Tedesco et al. [142] for the analysis of 20 carbohydrates in honeys, enabled researchers to detect analytes in the range of concentrations from several to several hundred µg/L. The LOD values revealed an increasing tendency in the following series: sucrose (5 µg/L) < glucose, galactose (6 µg/L) < mannose (7 µg/L) < kojibiose, lactulose, ribose (8 µg/L) < arabinose, melezitose, raffinose, xylose (10 µg/L) < fructose, isomaltotriose, lactose, melibiose, nigerose (20 µg/L) < erlose (60 µg/L) < palatinose (90 µg/L) < turanose (100 µg/L) < stachyose (400 µg/L). Zhao et al. [93] made a comparative analysis of carbohydrate determination methods in spirulin using various detectors. They demonstrated that, if identical chromatographing parameters were maintained, the HPAEC-PAD method featured lower values of LODs for saccharides, mannitol and sugar acids from the studied samples than the HPAEC-MS method (LOD = 0.02–0.10 µg/L vs. 0.2–1.5 µg/L, respectively). This was not the case for sucrose, for which the LOD was equal in both cases, amounting to 0.02 µg/L.

## 5. Conclusions

Millions of analyses for all types of analytes, including carbohydrates, are carried out all around the world every day. It is necessary, first and foremost, due to regulations in force on assuring both the quality of environment and food. Unfortunately, these actions have still been generating high costs, which is associated with the number of analyses conducted, the use of toxic chemical reagents, the produced waste and the consumed energy. Therefore, there has been a growing interest in the pro-ecological aspects of the implemented analytical methods. One of them is IC, because applied eluents are relatively inexpensive, safe to use, environmentally friendly, and only a small amount of waste is generated (typically nontoxic).

Nowadays, a number of methods for carbohydrate determination, including IC and related techniques, are available for chemists’ use. All of them are characterized by specific advantages and limitations. IC and related techniques may constitute an interesting complementary asset both for commercial and scientific laboratories involved in routine analyses, as well as for those that would like to broaden their analytical and commercial potential. Over the recent years, IC has achieved a high technological level due to the introduction of novel stationary phases, innovative methods of suppression and sample preparation, as well as the analysis methods themselves (capillary and multidimensional techniques). On that basis, it is now possible to use IC also for analysis of substances that, under defined conditions, may form ionic forms, just like carbohydrates.

This paper reviews the uses of IC and related techniques in the determination of selected carbohydrates and their derivatives for samples of air as well as example plants, fruits and vegetables. There are two fundamental reasons for such a choice. The first is the condition of the environment, which exerts a direct effect on the quality of grown plants and harvested crops. The second stems from the first and is manifested by the quality of food consumed by humans, and, ultimately, by our health. The scope of qualitative and quantitative carbohydrate studies presented in the literature is impressive. That gives positive evidence not only as to the need for determining this particular group of analytes in various matrices, but also as to possibilities of IC and relative techniques within the applicatory range discussed here. Literature examples from the last decade, cited in this paper, unambiguously demonstrate that these techniques, utilizing isocratic or gradient elution assisted by amperometric detection, is a useful analytical method involved in carbohydrate determination. Its popularity results from the availability, versatility, beneficial validation parameters including detection limits, relatively low costs of the analyses, and environmental aspects. The importance of IC and related techniques in carbohydrate research are unquestionable. IC will continue to evolve as more ionic contaminants become regulated, and not only in environmental and food research.

## Figures and Tables

**Table 1:** Examples of commercially available ion exchange columns used for the determination of carbohydrates (PS—polystyrene; DVB—divinylobenzene; EVB—ethylvinylbenzene; VBC—vinylbenzyl chloride; TMA—trimethylammonium).

Column Name	CarboPac PA1	CarboPac PA10	CarboPac PA20	CarboPac PA100	CarboPac MA1	CarboPac PA200	CarboPac SA10	CarboPac PA210- Fast-4 μm	Metro-sep Carb2	RCX-30
Manufacturer	Dionex	Dionex	Dionex	Dionex	Dionex	Dionex	Dionex	Dionex	Metrohm (Herisau, Switzerland)	Hamilton (Giarmata, Romania)
Column dimensions [mm]	250 × 4.0	250 × 4.0	250 × 4.0	250 × 4.0	250 × 4.0	250 × 3.0	250 × 4.0	150 × 4.0	250 × 4.6	250 × 4.6
pH range	0–14	0–14	0–14	0–14	0–14	0–14	0–14	0–14	0–14	1–13
Filling	PS/ DVB	EVB/ DVB	EVB/ DVB	EVB/ DVB	VBC/ DVB	EVB/ DVB	EVB/ DVB	EVB/ DVB	PS/ DVB	PS/ DVB/ TMA
Ion exchange capacity [µval/column]	100	65	65	90	1450	35	290	66	1530	2000
Particle size [μm]	10	10	6.5	10	8.5	8.5	6.0	4.0	5	7
Cross-linking [%]	5	5	5	6	15	6	5	6	-	-
Recommended eluent flow [mL/min]	1	1	5	1	0.4	0.5	1.5	0.8	0.5	1.0
Maximum preasure [MPa]	27.5	27.5	24.1	27.5	9.6	18.6	24.1	27.5	20	34
Compatibility with organic solvent [%]	2	90	100	100	0	100	100	100	0–50	-

**Table 2:** Examples of carbohydrate determination in atmospheric samples, taking into account analytical conditions.

Matrix	Analyte	Column/Precolumn	Elution Mode	Elution Parameters	Detection Mode	Ref.
PM1	glucose, fructose, mannose, galactose, sucrose, levoglucosan, mannosan, galactosan, arabitol, mannitol, erythritol	CarboPac MA1 (4 × 250 mm)/(4 × 50 mm)	Gradient: −15–−14.9 min: 35% A + 65% B; 14.9–20 min: 52% A + 48% B; 20.1–45 min: 35% A + 65% B	A: H_2_O; B: 1.0 M NaOH; 0.4 mL/min; room temperature; 25 µL	HPAE-PAD	[59]
PM2.5	levoglucosan, arabitol, mannitol, erythritol, xylitol, glycerol, myo-inositol, glucose, mannopyranose, trehalose, mannose, galactose	CarboPac MA1 (4 × 250 mm)/(4 × 50 mm)	Gradient: 200–600 mM A	A: NaOH; B: H_2_O; 0.4 mL/min; 400 mL	HPAEC-PAD	[60]
PM10						[61]
PM 2.5	levoglucosan	CarboPac PA20	Gradient: 0–15 min: 18 mM; 15–40 min: 18–200 mM; 40–50 min: 200 mM	NaOH	HPAEC-PAD	[62]
PM2.5	levoglucosan	CarboPac PA10 (2 × 250 mm)/(2 × 250 mm)	Gradient: 0–15 min: 0.5 mM; 0.5–10 mM (2.375 mM/min); 10–50 mM (4 mM/min); 65 mM for 6 min; 0.5 mM for 4 min	KOH; 0.20 mL/min	HPAEC-MS	[63]
PM1, PM10	levoglucosan, mannosan, galactosan, inositol, xylitol, sorbitol, mannitol, glucose	CarboPac PA10 (4 × 250 mm)	Isocratic: 0–25 min: 25 mM; cleaning: 200 mM for 8 min; reequilibration: 25 mM for 17 min	NaOH; 0.5 mL/min	HPAEC-PAD	[64]
Marine and atmospheric samples	fucose, rhamnose, arabinose, galactose, glucose, mannose, xylose, fructose, ribose, xylitol, arabitol, sorbitol, mannitol, levoglucosan, mannosan, galactosan, sucrose	Set 1.CarboPac MA1 (7.5 μm, 4 × 250 mm)/(4 × 50 mm) Set 2. CarboPac PA1 (10 μm, 4 × 250 mm)/(4 × 50 mm)	Set. 1. Gradient: 0–30 min: 250–350 mM NaOH; 30–45 min: 350–450 mM NaOH; 45–55 min: 450–700 mM NaOH; 55–75 min: 700 mM NaOH; 75–95 min: 250 mM NaOH; Set 2. 0–15 min: 1 mM NaOH; 15–38 min: 19 mM NaOH	Set 1. NaOH; 0.3 mL/min; detector: 25 °C, column: 0–30 min: 25–28 °C; 30–75 min: 28 °C; 75–95 min: 25 °C; 230 µL; Set 2. NaOH; 0.7 mL/min, 17 °C (column); 20 °C (detector)	HPAEC-PAD	[65]
PM10	glucose, arabitol, mannitol	Set 1: A Supp 15–150 + Carb 1–150/Carb 1-Guard; Set 2: Carb (2 × 150 mm)/(2 × 50 mm). Set 3: CarboPac MA1 (4 × 250 mm)	Isocratic: Set 1: 120 mM NaOH for 9 min (cleaning); 70 mM NaOH for 11 min, NaOH for 9 min. Set 2: 15% B + 85% C; Set 3: 480 mM NaOH	A: NaOH; B: 200 mM NaOH + 4 mM NaOAc; C: H_2_O; 1 mL/min; room temperature; 25 µL	HPLC-PAD	[66]
PM2.5	levoglucosan, mannnosan, galactosan	CarboPac PA1 (10 µm, 2 × 250 mm)/(10 µm, 2 × 50 mm)	Gradient: 0–2 min: 2 mM; 2–7 min: 2–10 mM; 7–10 min: 10–200 mM; 10–11 min, 200 mM; 11–11.1 min: 200–2 mM; 11.1–15 min: 2 mM	NaOH; 0.25 mL/min; 30 °C; 10 μL	HPAEC-positive ESI-MS	[67]
Ambient aerosols	levoglucosan	CarboPac PA10 (2 × 250 mm)/(2 × 50 mm)	I: Isocratic; 2 mM; II: 0.5 mM for 1 min; 0.5–10 mM (2.375 mM/min); 10–50 mM (4 mM/min); 50 mM for 3 min; 0.5 mM for 7 min; III: 0.5 mM for 1 min; 0.5–10 mM (2.375 mM/min); 10–50 mM (4 mM/min); 65 mM for 6 min; 0.5 mM for 4 min	KOH; I. 0.25 mL/min; II: 0.20 mL/min; III: 0.20 mL/min	PILS-HPAEC-MS	[68]
Antarctic aerosol	arabinose, fructose, galactose, glucose, mannose, ribose, xylose, sucrose, lactose, lactulose, erythritol, maltitol	Set 1. CarboPac PA10 (2 × 250 mm)/(2 × 50 mm) and AminoTrap (2 × 50 mm); Set 2. CarboPac MA1 (2 × 250 mm)/AminoTrap (2 × 50 mm)	Gradient: Set 1. 0–3 min: 1 mM; 3–20 min: 10–20 mM; 20–45 min: 20 mM; 45–55 min: 100 mM; 55–60 min: 1 mM; Set 2. 1, 0–23 min: 20 mM; 23–43 min: 100 mM; 43–53 min: 20 mM	NaOH; 0.25 mL/min; 50 μL	HPAEC-MS	[69]
PM10	arabinose, fructose, galactose, glucose, mannose, ribose, xylose, sucrose, arabitol, erythritol, mannitol, ribitol, sorbitol, xylitol, galactitol, maltitol, levoglucosan, mannosan, galactosan					[70]
Antarctic aerosols (TSP)	levoglucosan, galactosan, mannosan, arabinose, ribose, xylose, fructose, galactose, glucose, mannose, sucrose, erythritol, arabitol, xylitol, ribitol, mannitol, sorbitol, maltitol					[71]
PM10	glucose, fructose, trehalose, sucrose, cellobiose, galactose, erythritol, inositol, mannitol, arabitol, galactosan, mannosan, levoglucosan	CarboPac MA1 (4 × 250 mm)/(4 × 50 mm)	Gradient: -0–20 min: 480 mM; 35–45 min: 650 mM; reequilibration: 450 mM for 14.9 min	A: H_2_O; B: 1 M NaOH; 0.4 mL/min; 25 °C; 25 μL	HPAEC-PAD	[72]
PM2.5	levoglucosan, mannosan, galactosan, glucose, mannose, erythritol, arabitol, mannitol	CarboPac MA1 (4 × 250 mm)/(4 × 50 mm)	Isocratic	250 mM NaOH; 0.5 mL/min	HPAEC-PAD	[73]
PM2.5	levoglucosan					[74]
PM10	xylitol, arabitol, mannosan, trehalose, mannitol, levoglucosan, galactosan, glucose, galactose, fructose, sucrose	CarboPac MA1 (4 × 250 mm)/(4 × 50 mm)	Gradient: 0–20 min: 480 mM; 35–45 min: 650 mM; reequilibration: 480 mM for 14.9 min	A: H_2_O; B: 1 M NaOH; 0.4 mL/min; 25 °C; 25 μL	HPAEC-PAD	[75]
PM10	levoglucosan					[76]
Atmospheric samples (terrestial, marine, rain)	levoglucosan, mannosan, galactosan	CarboPac RPA-1 (10 μm, 2 × 250 mm)/(2 × 50 mm)	Gradient: 9 min cleaning; 0–2: 0.5 mM; 2–7 min: 0.5 –10 mM; 7–9 min: 10–100 mM; 9–15 min: 100 mM; 15–17 min: 100–0.5 mM; 17–20 min: 0.5 mM;	KOH; 0.25 mL/min; 30 °C; 50 μL 20 min	IC-TSQ-MS	[77]

**Table 3:** Examples of the determination of carbohydrates in biomass, taking into account analytical conditions.

Matrix	Analyte	Column/Precolumn	Elution Mode	Elution Parameters	Detection Mode	Ref.
Beechwood	xylose, xyobiose, xylotriose, xylopentaose, xylohexaose	CarboPac PA200 (3 × 250 mm)/AminoTrap (3 × 50 mm)	Gradient: 0–30 min: 2 to 150 mM B 30–60 min: B + A	A: 150 mM NaOH B: NaOAc; 35 °C; 20 μL; 0.45 mL/min	HPAEC-QqQ-MS	[78]
Beechwood (*Fagus sylvatica* L.)	glucose, arabinose, xylose, glucuronic acid					[79]
Beechwood	fucose, glucose, xylose, galactose, mannose, rhamnose, arabinose, galacturonic acid, glucuronic acid	CarboPac PA20 (3 × 150 mm)	Gradient: 0–20 min: 0.8% A + 99.2% B; 20 –37 min: 20% A + 75% B + 5% C; 37–41 min: 20% A + 40% B + 40% C	A: 250 mM A; B: H_2_O; C: 1 M NaOAc in 20 mM NaOH; 0.4 mL/min; 35 °C; 45 min; 20 μL	HPAE-PAD	[80]
Hardwood *Aucoumea klaineana*	xylose, mannose, glucose, arabinose, galactose, rhamnose, galacturonic acid, glucuronic acid	CarboPac PA20 (3 × 150 mm/(3 × 30 mm)	Gradient: A + B + C in variable proportion	A: 250 mM A; B: H_2_O; C: 1 M NaOAc; 0.4 mL/min; 35 °C	HPAEC-PAD	[81]
			Gradient: 0–20 min: 0.8% A + 99.2% B; 20–37 min: 20 % A + 75% B + 5% C; 37–45 min: 20% A + 40% B + 40% C	A: 250 mM A; B: H_2_O; C: 1 M NaOAc + 20 mM NaOH; 0.4 mL/min; 35 °C; 45 min		[82]
Aspen wood chips	glucose, arabinose, mannose, galactose	CarboPac PA20 (3 × 150 mm)/(3 × 30 mm)	Gradient: 0–22 min: 4% A + 96% B; 22–27 min: 40% A + 20% B + 40% C; 28–35 min: 20% B + 80% D	A: 50 mM NaOH; B: H_2_O µL; C: 1 M NaOAc; D: 50 mM NaOH; 0.4 mL/min; 30 °C (column) and 25 °C (detector)	HPAEC-PAD	[83]
						[84]
Pine wood pyrolysis oils	levoglucosan, arabinose, galactose, glucose, xylose	CarboPac PA20	Isocratic	80% H_2_O + 20% 50 mM NaOH; 0.50 mL/min; 10 μL; 35 °C	HPAEC-PAD (detector pH = 10.4)	[85]
Spruce and beech pulps, Wood: *Picea abies* (L.) *Eucalyptus globulus*, *Fagus sylvatica* L., *Quercus alba* L., *Alnus glutinosa* (L.), *Populus alba* L., wheat straw and bagasse	xylose, galactose, glucose, arabinose, mannose, MeGlcA-xylose MeGlcA, galacturonic acid	CarboPac PA200 (3 × 250 mm)/(3 × 50 mm)	Gradient: 30 min: 7.5% B 30–60 min: up to 60% B; 60–85 min: 60% B; cleaning: 200 mM NaOH for 20 min; equilibration: 7.5% B for 10 min	A: H_2_O, B: 1 M NaOAc in 200 mM NaOH; 0.4 mL/min; 10 μL; 30 °C	HPAEC-UV	[86]
Rice hull	arabinose, galactose, glucose, mannose, xylose	CarboPac PA1	isocratic	10 mM NaOH + 1 mM (CH_3_COO)_2_Ba; 1 mL/min	HPAEC-PAD	[87]
Sugar cane bagasse, wheat and barley straw, willow wood	glucose, xylose galactose, arabinose, mannose, ribose, cellobiose, xylobiose, arabinobiose, mannobiose, cellotriose, xylotriose, mannotriose, xylotetraose, galactonic acid, gluconic acid, galacturonic acid, glucuronic acid, lactobionic acid	CarboPac PA1 (2 × 250 mm)	Gradient: 0–5 min: 100% A, 5–78 min: linear gradient from 100–74% (linear); 100% B for 6 min (cleaning); 100% A for 10 min (re-equilibration)	A: 100 mM NaOH; B: 100 mM NaOH + 500 mM NaOAc. 215 µL/min; 3 µL; 35 °C	HPAEC-MS	[88]
Chardonnay grape marc	hexose, deoxyhexose, hexuronic acid, pentose, pentose alditol	CarboPacPA300-4 μm (2 × 250 mm)/(2 × 50 mm)	Gradient: 0–15 min: 68.5–5% A; 29.5–35% B; 2–60% C; 15–45 min: 5–25% A; 35–25% B; 60–0% C; 0–50% D; 45–55 min: 25–0% A; 25–0% B; 50–100% D; 55–55.9 min: 100% D; 60–75 min: 68.5% A; 29.5% B; 2% C	A: H_2_O; B: 200 mM NaOH; C: 25 mM NaOAc in 50 mM NaOH; D: 250 mM NaOAc in 100 mM NaOH; 0.25 mL/min; 10 μL (partial loop); 4 °C	IC-PAD/MS/MS	[89]
Sugarcane bagasse	arabinose, galactose, glucose, xylose	CarboPac PA 10 (4 × 250 mm)/(4 × 50 mm)	Isocratic	99% H_2_O + 1% 150 mM NaOH; 1 mL/min; 25 °C (column); 35 °C (detector); 25 μL	HPAEC-RPAD	[90]
Sugarcane bagasse	arabinose, galactose, glucose, xylose, mannose	CarboPac PA20	Isocratic	8 g/L NaOH + 20 g/L NaOAc; 0.3 mL/min; 25 µL; 30 °C	HPAEC-PAD	[91]
Sugarcane bagasse pith	arabinose, galactose, glucose, xylose, glucuronic acid, galacturonic acid	CarboPac PA1	Isocratic	8 g/L NaOH + 20 g/L NaOAc; 1 mL/min; 25 µL; 30 °C	HPAEC-PAD	[92]

**Table 4:** Examples of the determination of carbohydrates in plants, taking into account analytical conditions.

Matrix	Analyte	Column/Precolumn	Elution Mode	Elution Parameters	Detection Mode	Ref.
*Spirulina platensis*	fucose, fructose, rhamnose, arabinose, galactose, glucose, xylose, mannose, ribose, mannitol, sucrose, galacturonic acid, glucuronic acid	CarboPac PA20 (3 × 150 mm)/(3 × 50 mm)	Gradient: 0–5 min: 30–60% B, 0–20% C; 5–15 min: 60–20% B, 20–80% C; 15–25 min: 20–30% B, 80–20% C; 25–25.1 min: 30% B; 20% C; 25.1–30 min: 30% B, 20–0% C	A: H_2_O; B: 0.1 M NaOH; C: 0.4 M NaOAc; 0.45 mL/min; 30 °C; 25 μL	HPAEC-PAD-MS	[93]
*Posidonia oceanica*, *Ascophyllum nodosum*	adonitol, mannitol, fucose, arabinose, galactose, glucose, xylose, mannose	CarboPac PA20 (3 × 150 mm)/(3 × 30 mm)	Gradient: 0–21 min: 1.5% B; 21–33 min: 50% B; 33–49 min: 100% C; 49–53 min: 100% A; 53–70 min: 1.5% B	A: H_2_O Milli-Q; B: 0.2 M NaOH; C: 0.1 M NaOH + 0.1 M NaOAc; 0.4 mL/min; 30 °C	HPAEC-PAD	[94]
*Mesona chinensis benth*	glucose, mannose, xylose, rhamnose, arabinose, galactose, fructose, ribose, fucose	CarboPac PA10 (2.0 × 250 mm)	Isocratic	12.5 mM NaOH; 0.25 mL/min; 25 μL; 30 °C	HPAEC-PAD	[95]
*Mesona chinensis benth*	rhamnose, arabinose, galactose, glucose, xylose, mannose, glucuronic acid, galacturonic acid	CarboPac PA20 BioLC (3 × 150 mm)/(3 × 30 mm)	Isocratic	250 mM NaOH + H_2_O + 1 M NaOAc	HPAEC-PAD	[96]
*Mesona chinensis* herbaceous plant	glucose, rhamnose, arabinose, fucose, xylose, mannose, galactose	Carbo PAC PA10 (2.0 × 250 mm)	Isocratic	12.5 mM NaOH; 0.25 mL/min; 25 μL; 30 °C	HPAEC-PAD	[97]
Sweet tea tree *Cyclocarya paliurus*	arabinose, rhamnose, fucose, fructose, xylose, galactose, glucose, mannose					[98]
Lupine seeds (*Lupinus angustifolius*	galactose, glucose, sucrose, fructose, raffinose, stachyose, verbascose, maltose, 2-deoxy-d-glucose	CarboPac PA100 (2 × 250 mm)/(2 × 50 mm)	Gradient: 10% A at 0 min, 25% A at 10 min, 25% A at 12 min, 95% A at 52 min, 95% A at 56 min, 10% A at 57 min, 8 min at 10% A (equilibration)	A: 0.145 M NaOH; B: H_2_O; 0.25 mL/min; 1 μL; 68–73 min	HPAEC-PAD	[99]
Iridaceae species	glucose, fructose, sucrose, raffinose, maltose, 1-kestose, nystose, neokestose	CarboPac PA100 (4 × 250 mm)	Gradient: 0–2 min: 5 mM; 2.1–8 min: 5–50 mM; 8.1–11 min: 50–150 mM; 11.1–14 min: 250 mM; 14.1–18 min: 5 mM	NaOAc in 150 mM NaOH; 1 mL/min	HPAEC-PAD	[100]
Red deadnettle (*Lamium purpureum*, Lamiaceae)	glucose, fructose, melibiose, manninotriose, sucrose, raffinose, stachyose, galactose, galactinol, mannitol	CarboPac PA100	Gradient: 90 mM NaOH for 9 min (equilibration); 0–6 min: 0–10 mM A; 6–16 min: 10–100 mM A; 500 mM NaOAC for 5 min (regeneration)	A: NaOAc; 1 mL/min;	HPAEC-PAD	[101]
*Opuntia joconostle*	rhamnose, arabinose, xylose, galactose, fucose, glucose, galacturonic acid	CarboPac PA1 (4 × 250 mm)/(4 × 50 mm)	Gradient: −20 to −2.5 min: A; −2.5–22 min: 10 mM NaOH + 2 mM NaOAc; 22–40 min: ramp up to B; 40–50 min: A	A: 200 mM NaOH; B: 200 mM NaOH + 200 mM NaOAc; C: H_2_O; 1 mL/min; 30 °C; 10 µL	HPAEC-PAD	[102]
*Opuntia ficus-indica*, *Opuntia joconostle*	rhamnose, arabinose, xylose, galactose, glucose, galacturonic acid					[103]
Roselle (*Hibiscus sabdariffa* L.)	glucose, fructose, sucrose	Metrosep CARB 1 (4.0 × 150 mm)	Isocratic	100 mM NaOH; 1.0 mL/min; 20 μL; 35 °C	IC-PAD	[104]
*Laminaria japonica* Fucoidans	fucose, galactose, glucose, mannose, rhamnose, xylose	CarboPac PA10 (4 × 250 mm)	Isocratic	200 mM NaOH; 0.5 mL/min	HPAEC-PAD	[105]
*Astragalus membranaceus*	fucose, rhamnose, arabinose, galactose, glucose, xylose, mannose, glucuronic acid, galacturonic acid	CarboPac PA20 (3 × 150 mm)	Isocratic	6 mM NaOH + 100 mM NaOAc; 1 mL; 30 °C;	HPAEC-PAD	[106]
*Laminaria digitata*, *Saccharina latissimi*	fucose, rhamnose, arabinose, galactose, xylose, mannose, galacturonic acid, glucuronic acid; glucose, xylose	Set 1. CarboPac PA20 Set 2. Omnifit bore (6.6 × 115 mm) filled with resin MCI Gel CA08F resin	Set 1. 25 min: 1% B in A for 25 min; 200 mM NaOH (0.2 mL/min) for 25 min; 200–20 mM NaOH for 25 min (linear). Set 2. 90% A + 10% B; to 10%A + 90%B for 35 min (linear).	Set 1. A: H_2_O, B: 200 mM NaOH; 1 M NaOAc in 200 mM NaOH; 0.4 mL/min; 25 min; Set 2. A: 0.3 M potassium borate buffer pH 9.2; B: 0.9 M potassium borate buffer pH 9.2; 0.7 mL/min	Set 1. HPAEC-PAD; Set 2. borate-HPAEC-UV/Vis	[107]
*Agave* *angustifolia* Haw., *Agave potatorum* Zucc.	glucose, fructose, sucrose, 1-ketose, nystose, fructosyl-nystose	CarboPac PA100 (4 × 250 mm)/(4 × 50 mm)	Gradient: 0–5 min: 45 mM A; 5–60 min: 0–375 mM B; 60–65 min: 500 mM B; and 65–75 min: 45 mM A	A: NaOH; B: NaOAc in 0.15 M NaOH; 0.8 mL/min; 25 μL; 25 °C	HPAEC-PAD	[108]
*Agave tequilana*	glucose, fructose, sucrose; inulin; fructooligosaccharides	CarboPac PA100 (4 × 250 mm)/(4 × 50 mm)	Gradient: 0 to 500 mM A	A: NaOAc in 0.15 M NaOH; 0.8 mL/min; 25 μL; 25 °C	HPAEC-PAD	[109]
Wild Agave varieties	glucose, fructose, sucrose, kestose, nystose	CarboPac PA100 (40 × 250 mm)/(40 × 25 mm)	Gradient	A: 100 mM NaOH B: 600 mM NaOAc; 35 °C	HPAEC-PAD	[110]
*Eremurus hissaricus*	glucose, mannose, arabinose galactose, xylose, rhamnose, fucose, glucuronic acid, galacturonic acid	CarboPac PA20	Gradient: 1 mM A for 15 min, 0–130 mM B for 30 min; 1.0 M NaOAc for 0.8 min (cleaning); 1 mM NaOH for 30 min (re-equilibration)	A: NaOH, B: NaOAc in 100 mM NaOH; C: H_2_O; 0.5 mL/min	HPAEC-PAD	[111]
*Sesamum radiatum* Schumach. &Thonn.	glucuronic acid, mannose, galactose, xylose, glucose, rhamnose, arabinose	CarboPac PA1 (4 × 250 mm	25 mM NaOH; gradient of NaOH and NaOAc	A: NaOH, B: NaOAc; C: H_2_O; 1 mL/min; 25 µL; 30 °C	HPAEC-PAD	[112]
*Agave angustifolia*	fructans	CarboPac PA100 (4 × 250 mm)/(4 × 50 mm)	Gradient: 0 to 500 mM A	A: NaOAc in 0.15 M NaOH; 0.8 mL/min; 25 μL; 25 °C	HPAEC-PAD	[113]
Phyllostachys Pubescens	glucose, xylose, fructose, galactose, arabinose	Hamilton RCX-30 (7 μm; 4.6 × 250 mm)	Isocratic	2.0 mM NaOH and 0.5 mM NaOAc; 1 mL/min; 2 mL; 60 min	HPAEC-PAD	[114]
Tea seeds	fucose, mannose, xylose, ribose, glucose, galactose, rhamnose, arabinose, galactose acid, glucose acid	CarboPac PA20 (3 × 150 mm)	Isocratic	2 mM NaOH; 0.45 mL/min; 25 μL; 30 °C	HPAEC-PAD	[115]
Peach kernel	glucose, fructose, sucrose	CarboPac PA100 (4 × 250 mm, 8.5 μm, <10 Å)	Gradient: linear, 0–5 min: 15% A + 85% C; 5.0–5.1 min,: 15% A + 2% B + 83% C; 5.1–12.0 min: 15% A + 2% B + 83% C; 12.0–12.1 min: 15% A + 4% B + 81% C; 12.1–20.0 min: 15% A + 4% B + 81% C; 20.0–20.1 min: 20% A + 20% B + 60% C; 20.1–30.0 min: 20% A + 20% B + 60% C; preconditioning with 15% A + 85% C for 15 min	A: 600 mM NaOH; B: 500 mM NaOAc; C: H_2_O; 0.7 mL/min; 25 μL; 30 °C	HPAEC-PAD	[116]
Jerusalem artichoke pectins	glucose, fructose, galactose, rhamnose, mannose, maltose, arabinose, xylose	CarboPac PA1 (2 × 250 mm) with precolumn (2 × 50 mm)	Gradient: 10 mM NaOH (0–25 min); 100 mM NaOH (25–45 min)	NaOH; 0.30 mL/min; 25 μL; 30 °C	HPAEC-PAD; HPAEC-ESI-MS	[117]
Alfalfa (*Medicago sativa* L.) plant	fucose, arabinose, galactose, glucose, xylase, mannose, fructose, ribnose, galacturonic acid, glucuronic acid	No data	Isocratic	200 mM NaOH or 200 mM NaOAc; 1.0 mL/min	IC-DAD	[118]
Tartary buckwheat seeds	maltose, starch, laminarin, xylan, avicel, cellobiose, lactose, sucrose	CarboPac PA20	Isocratic	6.25 mM NaOH; 0.5 mL/min	HPAEC-PAD	[119]

**Table 5:** Examples of determining carbohydrates in fruits, vegetables and mushrooms, taking into account analytical conditions.

Matrix	Analyte	Column/Precolumn	Elution Mode	Elution Parameters	Detection Mode	Ref.
Blackberry	fucose, rhamnose, arabinose, galactose, glucose, xylose, mannose, fructose, ribose, galacturonic acid, glucuronic acid, mannose acid, guluronic acid	CarboPac PA20 (3 × 150 mm, 10 μm)	Gradient: 0 min: A/B/C (95:5:0, *v*/*v*), 26 min: A/B/C (85:5:10, *v*/*v*), 42 min: A/B/C (85:5:10, *v*/*v*),42.1 min: A/B/C (60:0:40, *v*/*v*), 52 min: A/B/C (60:40:0, *v*/*v*), 52.1 min: A/B/C (95:5:0, *v*/*v*), 60 min: A/B/C (95:5:0, *v*/*v*)	A: H_2_O, B: 0.1 M NaOH, C: 0.1 M NaOH + 0.2 M NaOAc; 30 °C; 5 μL	HPEAC-PAD	[120]
Blueberry	trehalose, fructose, sucrose, maltose, glucose, maltotriose, gentiobiose, isomaltose	Carbo Pac PA100 (4 × 250 mm)	Gradient: 0–5 min: 5% A;5–11.9 min: 15% A + 2% B; 12–19.9 min: 15% A + 4% B + 81% C; 20.0–30 min: 20% A + 20% B + 60% C	A: 600 mM NaOH, B: 500 mM NaOAc, C: H_2_O	HPEAC-PAD	[121]
Blueberry and strawberry	trehalose, fructose, succrose, maltose, glucose, arabinose, maltotriose isomaltotriose, xylose, ribose, raffinose, melibiose, gentiobiose, isomaltose, panose					[122]
Bilberry (*Vaccinium myrtillus*)	glucose, fructose, sucrose, arabinose, galactose, xylose, *myo*-inositol	CarboPac PA20/Amino trap	Gradient: 9 to 100 mM KOH	KOH	HPEAC-PAD	[123]
Mulberry	galactose, glucose, arabinose	CarboPac PA1 (4 × 250 mm)	Gradient: 0–25 min: 1%; 25–40 min: 1% to 100% A (linear); 40–50 min: 100% A. For sugar acids: 0–20 min: 20% A; 20–30 min: 100% B, 30–45 min: 20 to 35% A (linear)	A: 500 mM NaOH; B: 100 mM NaOH + 170 mM NaOAc; 30 °C; 20 µL	HPEAC-PAD	[124]
Noni (*Morinda citrifolia* L.)	fucose, glucose, rhamnose, xylose, mannose, galactose, arabinose, glucuronic acid, galacturonic acid					[125]
Myrtle (*Myrtus communis* L.) fruit	rhamnose, arabinose, glucose, xylose, mannose, galactose, glucuronic acid, galacturonic acid.	CarboPac PA1 (4 × 250 mm)	Gradient: 0–30 min: 91 % A + 9% B; 30–35.1 min: 91% A + 7% B + 2% C; 35.1–50 min: 50% A + 50% C; 50. 1–65 min: 50% B + 50% C; 65.1–85 min: 100% C; 85.1–100 min: 100% B; 100.1–115 min: 91% A + 9% B	A: H_2_O; B: 200 mM NaOH; C: 200 mM NaOH in 1 M NaOAc; 1 mL/min; 17 °C	HPAEC-PAD	[126]
*Annona squamosa*	glucose, galactose, rhamnose, xylose, mannose, arabinose, glucuronic acid, galacturonic acid	CarboPac PA10 (4 × 250 mm)/(4 mm × 50 mm)	Isocratic	2 mM or 10 mM NaOH, 0.45 mL/min	HPAEC-PAD	[127]
	rhamnose, glucose, xylose, galactose, mannose, arabinose, glucuronic acid, galacturonic acid, p-nitrophenyl-α-d-glucopyranoside, acarbose					[128]
Oblačinska’ sour cherry	trehalose, rhamnose, arabinose, glucose, fructose, isomaltose, sucrose, melezitose, gentiobiose, turanose, isomaltotriose, maltose, panose, maltotriose, glycerol, erythritol, arabitol, sorbitol, galactitol, mannitol	CarboPac PA10 (4 × 250 mm)	Gradient: 0–20 min: 15% A; 20.1–30 min: 20% A; 0–5 min: 0% B; 5.1–12 min: 2% B; 12.1–20 min: 4% B; 20.1–30 min: 20% B	A: 600 mM NaOH; B: 500 mM NaOAc; C: H_2_O; 0.7 mL/min; 25 μL; 30 °C	HPAEC-PAD	[129]
Onion (*Allium cepa* L.)	fructose, glucose, sucrose, 1-kestose, nystose; (1)3-kestopentaose, (1)4-kestohexaose, (1)5-kestoheptaose, (1)6-kestooctaose, (1)7-kestononaose, *myo*-inositol	CarboPac PA200 (3 × 250 mm) with precolumn (3 × 50 mm)	Gradient: eluent B: 0–70 min: 27.5%; 70–75 min: 27.5–0%; 75–80 min: 0–66%; 80–80.01: 66–27.5%; 80.01–86 min: 27.5–66%; 86–90 min: 66%; 90–95 min: 66–27.5%; 95–110 min: 27.5%) and eluent C: 0–30 min: 2.5–30%; 30–60 min: 30–54%; 60–70 min: 54–72.5%; 70–75 min: 72.5–100%; 75–80 min: 100–34%; 80–80.01: 34–72.5%; 80.01–86 min: 72.5–34%; 86–90 min: 34%; 90–95 min: 34–2.5%; 95–110 min: 2.5%), with A to 100%.	A: H_2_O; B: 225 mM NaOH; C: 500 mM NaOAc; 0.25 mL/min; 5 µL; 25 °C; 110 min	HPAEC-PAD	[130]
Red spice paprika	trehalose, arabinose, glucose, fructose, sucrose, galactitol, ribose, maltose, xylose, rhamnose, mannose, raffinose, sorbitol	CarboPac PA10 (4 × 250 mm)	Gradient: 0.0–20.0 min: 15% A; 20.1–30.0 min: 20% A; 0.0–5.0 min: 0% B; 5.1–12.0 min: 2% B; 12.1–20.0 min: 4% B; 20.1–30.0 min: 20% B, 0.0–5.0 min, 85% C; 5.1–12.0 min: 83% C; 12.1–20.0 min: 81% C; 20.1–30.0 min: 60% C.	A: 600 mM NaOH, B: 500 mM NaOAc; C: H_2_O; 0.7 mL/min; 25 µL	HPAEC-PAD	[131]
Beans (*Phaseolus vulgaris*)	raffinose, stachyose, verbascose	CarboPac PA100 (4 × 250 mm)/(4 × 50 mm)	Isocratic	10 mM NaOH; 1.0 mL/min; 25 μL; room temperature	HPAEC/PAD (Au)	[132]
Beans (*Phaseolus vulgaris* L.)	xylose, fructose, mannose, galactose, glucose, sucrose, galacturonic acid, myo-inositol, arabitol,	IonPac PA20 (3 × 150 mm)	Isocratic	H_2_O; 100 μL (slit ratio of 50:50)	IC-ESI-MS	[133]
Chickpea	glucose, fructose, raffinose, stachyose, verbascose, sucrose, myo-inositol, galactinol,	Set 1. CarboPac PA200 (3 × 250 mm)/(3 × 50 mm) Set 2. CarboPac PA100 (4 × 250 mm)/(3 × 50 mm)	Gradient: Set 1. 0 min: 90% A + 10% B1; 0–15 min: to 20% A + 80 % B1; 15 min: to 90% A + 10% B1: 15–25 min: 90% A + 10% B1; Set 2. 0 min: 90% A + 10% B2; 0–25 min: to 100% B2; 25 min: to 90% A + 10% B2: 25–35 min: 90% A + 10% B2	Set 1. A: H_2_O; B1: 100 mM NaOH; 0.5 mL/min; Set 2. A: H_2_O; B2: 200 mM NaOH; 1.0 mL/min;	HPAEC–PAD	[134]
*Poria cocos* and *Atractylodes macrocephala*	trehalose, glucose, maltotriose, galacturonic acid	PEEK, 4 × 150 mm with PAMAM	Isocratic	10 mM NaOH; 1.0 mL/min; 25 μL	HPAEC-PAD	[34]
Strawberries	glucose, fructose, sucrose; 1-kestose, 1-nystose; 1-fructofuranosyl-d-nystose, raftilose, neoinulin type fructans, neonystose; neopentaose	CarboPac PA100 (4 × 250 mm)/(4 × 50 mm)	Gradient: 0 to 500 mM A	A: NaOAc in 0.15 M NaOH; 0.8 mL/min; 25 μL; 25 °C	HPAEC-PAD	[135]
Apples	glucose, fructose, sorbitol, sucrose, trehalose, maltose	CarboPac PA20 (3 × 150 mm)/(3 × 30 mm)	Isocratic	50 mM NaOH; 0.5 mL/min; 10 μL; 30 °C; 35 min	HPAEC-PAD	[136]
Banana (leaf, rhizome and fruit pulp)	glucose, fructose, sucrose, 1-kestotriose, inulobiose, 1,1-nystose, inulotriose, 1,1,1-kestopentaose, 1-kestotriose, 6-kestotriose, 6g-kestotriose, raffinose, stachyose, maltose, maltotriose	CarboPac PA100	Gradient: 90 mM NaOH for 9 min (equilibration); 0–6 min: 0–10 mM A; 6–16 min: 10–100 mM A; 500 mM NaOAc for 5 min (regeneration)	A: NaOAc; 1 mL/min;	HPAEC-IPAD	[137]
Shiitake mushrooms	galactose, glucose, xylose, fructose, ribose	CarboPac PA1	Gradient: 0~15 min: 5.00% A; 15~40 min: 5–30% A (linear); 40~45 min: 30 to 100% A (linear); 45~54 min: 100% A	A: 250.00 mM NaOH; 0.25 mL/min; 25 °C; 25 µL	HPEAC-PAD	[138]
Fungus *Auricularia polytricha*	fucose, rhamnose, arabinose, mannose, galactose, glucose, xylose, fructose	CarboPac PA20 (3 × 150 mm)	Gradient: 0–21 min: 98% A + 2% B; 21–21.1 min: to 93% A + 2% B + 5% C; 21.1–30 min: to 78% A + 2% B + 20% C; 30–30.1 min: to 20% A + 80% B; 30.1–50 min: 20% A + 80% B	A: H_2_O: B: 250 mM NaOH; C: 1 M NaOAc; 0.25 mL/min; 25 μL; 30 °C	HPAEC-PAD	[139]

## Data Availability

Not applicable.
